# Validation of an electronic coding algorithm to identify the primary indication of orthopedic surgeries from administrative data

**DOI:** 10.1186/s12911-020-01175-1

**Published:** 2020-08-12

**Authors:** John C. Giardina, Thomas Cha, Steven J. Atlas, Michael J. Barry, Andrew A. Freiberg, Lauren Leavitt, Felisha Marques, Karen Sepucha

**Affiliations:** 1 Center for Health Decision Science, Harvard T.H. Chan School of Public Health, 718 Huntington Avenue, Boston, MA 02115 USA; 2 Department of Orthopaedic Surgery, Massachusetts General Hospital, Boston, MA USA; 3 Harvard Medical School, Boston, MA USA; 4 Division of General Internal Medicine, Massachusetts General Hospital, Boston, MA USA

**Keywords:** Electronic medical record, Algorithm validation, Diagnostic code, Procedure code, Orthopedic surgery, Elective surgery, Shared-decision making

## Abstract

**Background:**

Determining the primary indication of a surgical procedure can be useful in identifying patients undergoing elective surgery where shared decision-making is recommended. The purpose of this study was to develop and validate an algorithm to identify patients receiving the following combinations of surgical procedure and primary indication as part of a study to promote shared decision-making: (1) knee arthroplasty to treat knee osteoarthritis (KOA); (2) hip arthroplasty to treat hip osteoarthritis (HOA); (3) spinal surgery to treat lumbar spinal stenosis (SpS); and (4) spinal surgery to treat lumbar herniated disc (HD).

**Methods:**

Consecutive surgical procedures performed by participating spine, hip, and knee surgeons at four sites within an integrated care network were included. Study staff reviewed electronic medical records to ascertain a “gold standard” determination of the procedure and primary indication status. Electronic algorithms consisting of ICD-10 and CPT codes for each combination of procedure and indication were then applied to records for each case. The primary measures of validity for the algorithms were the sensitivity and specificity relative to the gold standard review.

**Results:**

Participating surgeons performed 790 procedures included in this study. The sensitivity of the algorithms in determining whether a surgical case represented one of the combinations of procedure and primary indication ranged from 0.70 (HD) to 0.92 (KOA). The specificity ranged from 0.94 (SpS) to 0.99 (HOA, KOA).

**Conclusion:**

The electronic algorithm was able to identify all four procedure/primary indication combinations of interest with high specificity. Additionally, the sensitivity for the KOA cases was reasonably high. For HOA and the spine conditions, additional work is needed to improve the sensitivity of the algorithm to identify the primary indication for each case.

## Background

Administrative data are commonly used in orthopedics research, since the data allow investigators to gather information about large numbers of patients over time and analyze the relationship between diagnoses, procedures, costs, and outcomes [1]. This process relies on researchers’ ability to use administrative data to accurately identify patients with the clinical characteristics relevant to the study. This is not, however, a straightforward task, since administrative claims data are usually recorded for billing purposes, and are not necessarily well suited to make clinical determinations in a research setting [1].

This task is especially difficult for research that is focused on more subtle clinical differences, such as studies evaluating the use of shared decision-making (SDM) for orthopedic surgery decisions. Evaluating the use of SDM in the context of orthopedic surgery at a large scale is a current priority since clinical guidelines for hip and knee osteoarthritis and degenerative lumbar spine conditions recommend shared decision-making (SDM) to select appropriate patients for surgery [2–4]. SDM is most relevant when the optimal treatment decision will depend on the individual preferences and needs of the patient. For SDM research in orthopedics, the focus is not only on identifying whether a certain procedure was performed, but also on identifying whether it was performed for a specific clinical indication (e.g. total knee replacement to treat knee osteoarthritis) that is considered elective in that it is based on patient symptoms and functional impairment that have other potential treatment options (e.g., conservative treatment options, such as physical therapy).

Therefore, the ability to determine the specific condition indicating a surgical procedure is important for SDM research. For example, evaluating SDM would be relevant for a patient who received a total hip replacement to treat hip osteoarthritis, since the optimal treatment choice will often be determined by an individual patient’s preferences around the trade-off between continued hip pain and the risks of surgery. Evaluating SDM would be less relevant, however, for a patient receiving a hip replacement performed to repair a hip fracture, since individual preferences are less likely to determine the need for surgery. The ability to identify the specific indication for a surgical procedure is clearly important for SDM researchers and for evaluating the implementation of SDM performance measures by surgeons and hospitals.

### Rationale

To address the problem of identifying patients from administrative data in the context of research on orthopedic surgery, researchers have developed validated coding algorithms that link sets of diagnosis and procedure codes, such as *International Classification of Diseases* (ICD) diagnosis codes and the *Current Procedural Terminology* (CPT) procedure codes from the American Medical Association, to specific conditions and surgical procedures, and then compare the accuracy of those algorithms against a gold standard [5–15]. These existing algorithms, however, are not necessarily useful in the context of SDM research and other studies where the primary indication for surgery is also of particular interest.

Previous studies have validated algorithms to identify hip or knee arthroplasty procedures, such as Daneshvar, Forster and Dervin [12], but these studies generally only used ICD codes as part of the algorithm, as opposed to both ICD and CPT codes, and we could find no previous studies that validated an algorithm to identify the primary indication for hip or knee arthroplasty procedures using administrative data. Among previously published algorithms to identify spine surgeries, Cherkin et al. [15] validated an algorithm to identify patients with “mechanical low back problems,” which generally reflects an indication with multiple treatment options (i.e., fracture, infection, or a neoplasm were not included as an indication), but this study only used ICD-9 codes, rather than the current ICD-10 codes, and is old enough that it may not reflect current coding practices. Furthermore, other work has indicated that CPT codes, which Cherkin et al. [15] did not include in their algorithm, provide a greater level of detail about spine surgeries in administrative data [16]. Thus, no current algorithms exist to identify both (1) the primary indication for common orthopedic procedures and (2) whether a patient receiving surgery for that indication may have also been a candidate for conservative treatment options. The development of such an algorithm would allow SDM researchers to efficiently identify patients undergoing elective, first-time surgery who would be good candidates for retrospective SDM research and to identify trends in the implementation of SDM tools across a health system.

The purpose of this study is to examine the validity of an algorithm that uses both ICD-10 and CPT codes from an administrative claims database to identify patients receiving one of the following surgery and indication combinations where conservative treatment is often an option: (1) knee arthroplasty for knee osteoarthritis (KOA); (2) hip arthroplasty for hip osteoarthritis (HOA); (3) spinal surgery for lumbar spinal stenosis (SpS); and (4) spinal surgery for lumbar herniated disc (HD). The validity of the algorithm is determined by assigning classifications to a set of surgical cases, and then comparing these classifications with a “gold standard” chart review process.

## Methods

### Sample and data sources

The study involved surgical patients who were at least 21 years old (for spine surgery) and at least 40 years old (for hip and knee surgery) at four sites within an integrated health care network in eastern Massachusetts (two academic medical centers and two community hospitals). All surgical procedures performed by a selected set of spine, hip, and knee surgeons at these centers consecutively between June 1, 2018 and June 30, 2018 (for hip and knee surgeons) and June 1, 2018 and July 31, 2018 (for spine surgeons) were included in this algorithm validation study. The selected set of surgeons were affiliated with the orthopedic or neurosurgery departments at one of the four centers and had been previously identified by the chief of each surgery line for inclusion in a larger study on the use of SDM for orthopedic surgery. Overall, patients from 19 hip and knee surgeons and 16 spine surgeons were included in this study (one surgeon performed both types of surgery).

The set of surgical cases were identified by an automated search of each surgeon’s surgical schedule over the time periods listed above. For each case, two sets of data were collected: (1) visit notes, operative reports, lab results, and imaging reports for a comprehensive chart review; and (2) administrative clinical data for the automated algorithm. Both sets of data included all the information associated with the patient for the 90 days preceding surgery (inclusive of the surgery date). The administrative clinical data for the automated algorithm was drawn from a system-wide Research Patient Data Registry (RPDR) derived from billing data and electronic medical records (EMR), and consisted of the ICD-10 and CPT codes associated with each identified surgical patient over the 90 day timeframe [17]. We obtained approval for the use of the data in this study from the Institutional Review Board at Partners HealthCare (protocol 2005P002282).

### Automated algorithm

An algorithm mapping CPT and ICD-10 codes to each of the four conditions and procedures of interest was developed. The algorithm itself consists of two steps to classify each surgical case. First, the administrative data associated with each surgical case is searched for any of the inclusion CPT and ICD-10 codes listed in Table 1. If both an inclusion CPT and ICD-10 code are identified, the surgical case is classified as having a relevant procedure indicated by that condition (e.g., hip arthroplasty with HOA indication). If a patient only had a CPT code, only an ICD-10 code, or none of the inclusion codes listed in Table 1, the surgical case is classified as “other.” Additionally, if a spinal surgery case was classified with both an SpS and HD indication using the CPT and ICD-10 codes, the case is reclassified to have only one indication based on the patient’s age: an SpS indication is listed if the patient is 50 years or older and an HD indication is listed if the patient is younger than 50 years old.

**Table 1:** CPT and ICD-10 codes included in algorithm by combination of procedure and primary indication

Inclusion CPT Codes	Inclusion ICD-10 Codes (Require one or more three months prior to surgery)	Exclusion ICD-10 Codes (Three Months Prior to Surgery)
A: Spinal Surgery for Lumbar Spinal Stenosis		
22224, 22533, 22558, 22586, 22612, 22614, 22630, 22632, 22633, 22800, 22802, 22804, 22808, 22810, 22857, 22862, 22867, 63005, 63011, 63012, 63017, 63047, 63048, 63056, 63087, 63088, 63090, 63091, 63102, 63103, 63170	M48.00, M48.061, M48.062, M48.07, M48.08	G83.4, R15*, S32.0*, N31*, C41.2, M48.56*, M84.58*, R32, M48.46*, M84.48*, M84.40*, M84.60*, M48.57*, S22.08*
B: Spinal Surgery for Lumbar Herniated Disc		
22224, 22533, 22558, 22586, 22612, 22614, 22630, 22632, 22633, 22800, 22802, 22804, 22808, 22810, 22857, 22862, 22867, 62287, 63005, 63011, 63012, 63017, 63030, 63035, 63042, 63047, 63048, 63056, 63087, 63088, 63090, 63091, 63102, 63103, 63170	M51.06, M51.16, M51.17, M51.26, M51.27, M51.36, M51.37, M47.16, M47.26, M47.27	G83.4, R15*, S32.0*, N31*, C41.2, M48.56*, M84.58*, R32, M48.46*, M84.48*, M84.40*, M84.60*, M48.57*, S22.08*
C: Hip Arthroplasty for Hip Osteoarthritis		
27125, 27130, 27132	M16.0, M16.10, M16.11, M16.12, M16.2, M16.30, M16.31, M16.32, M16.4, M16.50, M16.51, M16.52, M16.6, M16.7, M16.9	M84.459*, M84.559*, S72.0*, S32.4*, M87*, M80*, M84.359*, M84.58*, S72.1*, S72.2*, C79.51, M84.30*, M84.40*, M84.48*, M84.50*, C41.4, C41.9
D: Knee Arthroplasty for Knee Osteoarthritis		
27438, 27440, 27441, 27442, 27443, 27445, 27446, 27447	M17.0, M17.10, M17.11, M17.12, M17.2, M17.30, M17.31, M17.32, M17.4, M17.5, M17.9	M80*, M87*, C79.51, C41.9

The asterisk (*) indicates that all children codes within the ICD-10 hierarchy of the listed code are also included in the algorithm

*Descriptions of each code are included in* Additional file 1

*CPT Copyright 2017 American Medical Association. All rights reserved. CPT® is a registered trademark of the American Medical Association*

Second, among the cases that were not classified as “other,” the data for the case is searched for any of the exclusion ICD-10 codes listed in Table 1. If none of these exclusion codes are found, the case is classified with the relevant condition of interest (i.e., SpS, HD, KOA, or HOA) as the primary indication for surgery. If at least one of the exclusion codes is found, the relevant conditions of interest are listed as a secondary indication and the primary indication is listed as “other.”

This process was automated using the R programming language (version 3.5.1) and the *dplyr* data manipulation package and was applied to the patient data associated with each of the surgical cases identified by the schedule review (as described in the Samples and Data Sources section) [18, 19]. Each algorithm was only applied to procedures conducted by surgeons on the relevant list (e.g., the KOA algorithm was only applied to cases from the list of knee surgeons) in order to provide a more relevant evaluation of the algorithms. The algorithm was initially applied to all surgeons included in the data set, but this artificially inflated specificity without changing sensitivity, since hip and knee surgeons are almost never assigned CPT codes associated with spine surgery, and vice versa. These results indicate, however, that the algorithm should still be applicable to administrative data that is not broken down by the type of procedure each surgeon performs.

#### Algorithm development

The ICD-10 and CPT codes that form the basis of the algorithm were selected in consultation with orthopedic surgeons and were refined on an ad hoc basis after comparing the codes to surgical cases that underwent operations from January 2018 to May 2018 at the four hospitals included in the study (to avoid “overfitting” the set of codes included in the algorithms, this training set of cases does not overlap with the validation set described above). During this refinement process, the most significant changes to the algorithms were the addition of new exclusion codes. In particular, an effort was made to identify all the ICD-10 codes related to fractures and neoplasms at the surgical site, which were often an indication for urgent, non-elective surgery in the reviewed training set.

### “Gold standard” chart review

The “gold standard” classification used to evaluate the validity of the algorithm was defined as the categorization of a surgical case after manual review of the patient’s EMR from the 90 days prior to surgery, including visit notes, operative report, lab results, and imaging studies. Each surgical case was reviewed by one of two randomly assigned staff members who recorded the following information (in consultation with a primary care physician, orthopedic surgeon, or other team members as needed): (1) if the type of surgery performed matched one of the procedures of interest (e.g., hip arthroplasty); (2) if any of indications for the surgery matched one of the diagnoses of interest (e.g., HOA), and (3) if that diagnosis was the primary indication for surgery, or if the procedure was performed primarily to treat another condition.

Specifically, the type of surgery performed was determined by a review of the operative report and coded into one of the following groups: (1) spinal surgery; (2) knee replacement; (3) hip replacement; (4) other. Then, the indications for surgery were determined by a review of the visit notes and imaging studies in the time prior to surgery and the primary diagnosis listed in the operative report. Using this review, the indications for surgery were coded into the following groups: (1) lumbar spinal stenosis; (2) lumbar herniated disc; (3) knee osteoarthritis; (4) hip osteoarthritis; (5) other. In order to differentiate between the SpS and HD indications, the inclusion criteria used by the Spine Patient Outcomes Research Trial (SPORT) were applied to the review of the imaging studies [20].

Finally, the primary indication for surgery was determined by a review of the initial surgical consult, pre-operative visits, and imaging study notes, along with the problem list recorded in the EMR. If SpS, HD, KOA, or HOA were listed as one of the indications, but were not the primary indication, the actual primary indication was coded into one of the following groups: (1) infection, (2) possible malignancy, (3) fracture, or (4) other.

Throughout this chart review process, staff consulted with an orthopedic spine surgeon, hip and knee arthroplasty surgeon, or an internal medicine physician whenever the classification of a surgical case was unclear or there was disagreement between reviewers, and a final determination was made. Additionally, an initial set of randomly selected training cases (*n* = 70) were reviewed by both staff members in order to ensure the reliability of the written protocol and training for this gold standard review process. The inter-rater reliability was high, with a Cohen’s kappa of 0.87. All subsequent reviews were primarily conducted by one staff member for each case, with consultation between team members as needed.

### Analysis

The primary measures of validity for the automated algorithms were the sensitivity and specificity of each of the four classification algorithms relative to the gold standard review. “Exact” Clopper-Pearson Confidence Intervals were also calculated [21]. Since both the algorithm and gold standard review classified cases on two different levels (i.e., condition is an indication vs. condition is the *primary* indication), two sets of sensitivity and specificity values were generated for each algorithm.

Specifically, a surgical case was considered a true positive if both the algorithm and gold standard review marked the case as having both (1) the procedure of interest (e.g., hip arthroplasty) and (2) the relevant condition (e.g., HOA) as either an indication or the primary indication for the procedure (depending on the level of classification being evaluated). Similarly, a case was considered a true negative if both the algorithm and gold standard review did not mark the case as having both a procedure of interest and the relevant condition as an indication.

In addition to the calculation of overall estimates of the sensitivity and specificity, a post hoc analysis of the misclassified cases was conducted to evaluate how the use of the algorithm might impact the external validity of a study that uses the algorithm to make eligibility determinations. The positive and negative predictive values of the algorithm were also calculated for different probabilities that any given surgical case is one that has the condition of interest as the primary indication for its respective procedure. This analysis was conducted to evaluate the usefulness of this algorithm in other settings with different surgical rates. All of the analyses were performed in the R programming language [18].

## Results

Across the four sites, there were 790 surgical cases identified during the study period. Table 2 shows the number of cases identified by the gold standard review for each of the procedure and indication combinations of interest, along with all other cases. Additionally, Table 2 lists the fraction of cases where the condition of interest was the primary indication for that procedure, also as evaluated by the gold standard. Note that this fraction is relatively high for all four indications, which suggests that once one of the four conditions is identified as an indication for the relevant surgery, it will likely be the primary indication. Many of the other surgeries (listed in the final column in Table 2), however, were also performed at the hip, knee, or lumbar spine, highlighting the fact that any algorithm used to identify the primary indication of a procedure must first also differentiate between the procedure of interest and all other orthopedic procedures. This is shown in Table 3, which breaks out the “Other Indication” column from Table 2 with the location of each of these surgical procedures – a large fraction of these procedures are also performed at the hip, knee, or lumbar spine.

**Table 2:** Distribution of surgical cases by indication, as determined by gold standard review

	Hip Arthroplasty/Hip OA	Knee Arthroplasty/Knee OA	Lumbar Spinal Surgery/Lumbar Spinal Stenosis	Lumbar Spinal Surgery/Lumbar Herniated Disc	Other Indication
Number of Surgeries	134	119	131	54	352
Average Age (Years)	62.5	67.3	66.9	52.5	58.3
Gender (Percent Female)	49.3%	62.2%	52.7%	53.7%	50.3%
Condition was Primary Indication (%)	128 (95.5%)	115 (96.6%)	127 (96.9%)	53 (98.1%)	NA^a^

*OA* = osteoarthritis

^a^Primary indications not determined for “Other” diagnoses

**Table 3:** Location of 352 Surgeries with an “Other” Indication, as determined by gold standard review

Surgery Location	Number of Procedures (%)
Ankle/Foot	17 (5%)
Arm/Hand	32 (9%)
Cervical Spine	71 (20%)
Femur	38 (11%)
Hip	50 (14%)
Knee	70 (20%)
Lumbar Spine^a^	52 (15%)
Thoracic Spine	22 (6%)

^a^These represented cases where neither lumbar spinal stenosis or herniated disc were an indication for the spinal surgery

Table 4 shows the results of the first step of the automated algorithm (i.e., identifying if a surgical case represented one of the four procedure/indication combinations of interest, whether or not the condition is the primary indication) compared against the results of the gold standard review. The two-by-two tables used to generate these results are available in Additional file 1.

**Table 4:** Results of First Step of Algorithm (Determining if a surgical case represents one of the four procedure/indication combinations of interest)

Procedure/Indication	Sensitivity (95% Confidence Interval)	Specificity (95% Confidence Interval)
Lumbar Spinal Surgery/Lumbar Herniated Disc	0.96 (0.87, 1.00)	0.74 (0.69, 0.79)
Lumbar Spinal Surgery/Lumbar Spinal Stenosis	0.91 (0.85, 0.95)	0.82 (0.77, 0.87)
Knee Arthroplasty/Knee Osteoarthritis	0.97 (0.92, 0.99)	0.84 (0.80, 0.88)
Hip Arthroplasty/Hip Osteoarthritis	0.99 (0.96, 1.00)	0.87 (0.83, 0.91)

*Note:* “Exact” Clopper-Pearson Confidence Intervals are used

Next, the results after the second and final step of the automated algorithm (i.e., identifying if a surgical case represented one of the four procedure/indication combinations of interest, with the condition as the primary indication for surgery) are compared with the results of the gold standard review in Table 5. Again, the two-by-two tables used to generate these results are available in Additional file 1.

**Table 5:** Results of Final Step of Algorithm (Determining if a surgical case represents one of the four procedure/indication combinations of interest, with the condition as the primary indication for surgery)

Procedure/Primary Indication	Sensitivity (95% Confidence Interval)	Specificity (95% Confidence Interval)
Lumbar Spinal Surgery/Lumbar Herniated Disc	0.70 (0.56, 0.82)	0.96 (0.94, 0.98)
Lumbar Spinal Surgery/Lumbar Spinal Stenosis	0.76 (0.68, 0.83)	0.94 (0.91, 0.97)
Knee Arthroplasty/Knee Osteoarthritis	0.92 (0.86, 0.96)	0.99 (0.98, 1.00)
Hip Arthroplasty/Hip Osteoarthritis	0.84 (0.76, 0.90)	0.99 (0.98, 1.00)

*Note:* “Exact” Clopper-Pearson Confidence Intervals are used

**Fig. 1:**
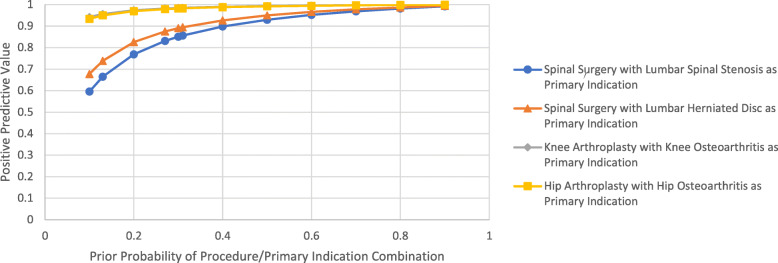
Positive Predictive Values of Algorithm for the Procedure/Primary Indication Combinations of Interest. The prior probabilities in the study sample were 0.31 for spinal surgery/spinal stenosis, 0.13 for spinal surgery/herniated disc, 0.27 for knee arthroplasty/knee osteoarthritis, and 0.30 for hip arthroplasty/hip osteoarthritis. Marks indicating the PPV for these prior probabilities are included in the figure

**Fig. 2:**
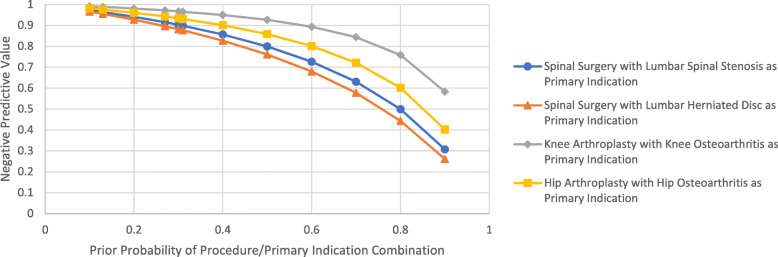
Negative Predictive Values of Algorithm for the Procedure/Primary Indication Combinations of Interest. The prior probabilities in the study sample were 0.31 for spinal surgery/spinal stenosis, 0.13 for spinal surgery/herniated disc, 0.27 for knee arthroplasty/knee osteoarthritis, and 0.30 for hip arthroplasty/hip osteoarthritis. Marks indicating the NPV for these prior probabilities are included in the figure

Following this final step of the algorithm, a set of positive and negative predictive values of the algorithm were calculated for each procedure/primary indication combination; the values are given for each combination in Figs. 1 and 2. Here, the given prior probability is the likelihood that any given surgical case from the sample of cases analyzed would be classified as that combination of procedure and primary indication by the gold standard review. For the sample analyzed in this study, the prior probabilities for the spinal surgery/SpS, spinal surgery/HD, knee arthroplasty/KOA, and hip arthroplasty/HOA combinations of procedure and primary indication were 0.31, 0.13, 0.27, and 0.30, respectively (as determined by the gold standard review).

Finally, Table 6 lists general reasons why particular surgical cases were “misclassified” by the algorithm after going through both steps of the algorithm. For the purposes of this paper, a misclassification is defined as a case where the algorithm did not have the same determination about the procedure and primary indication as the gold standard review did. For HD cases, the most common reason for misclassification was that the cases were classified by the gold standard review as SpS cases, and vice versa; this was likely because the algorithm used a strict age cutoff when a surgical case had the CPT and ICD-10 codes for both SpS and HD. Similarly, SpS cases were frequently misclassified because the gold standard classification was HD, or vice versa. Additionally, SpS cases were often misclassified because the procedure was actually performed on the cervical or thoracic spine, and because the algorithm did not include the CPT codes listed with an SpS procedure (or the data source did not include a comprehensive record of the CPT codes associated with the procedure).

**Table 6:** Reason for Algorithm Misclassification

Diagnosis	Misclassification	Reasons
Lumbar Herniated Disc (*n* = 29)	False Positive (*n* = 13)	Spinal Stenosis Case (*n* = 8) Severe Incontinence Diagnosis (*n* = 1) Cervical or Thoracic Spine Case (*n* = 1) Flatback Syndrome Case (*n* = 1) Spondylolisthesis Case (*n* = 1) Revision Discectomy Case (*n* = 1)
	False Negative (*n* = 16)	Incorrectly Classified as Spinal Stenosis (*n* = 9) Procedure Billing Codes not included in Data (*n* = 4) Incorrectly Marked as Non-Primary Indication because of urinary incontinence diagnosis (*n* = 2) Incorrectly Marked as Non-Primary Indication because of cauda equina diagnosis (*n* = 1)
Lumbar Spinal Stenosis (*n* = 46)	False Positive (*n* = 16)	Herniated Disc Case (*n* = 9) Cervical or Thoracic Spine Case (*n* = 4) Infection Present (*n* = 1) Flatback Syndrome Case (*n* = 1) Epidural Abscess Case (*n* = 1)
	False Negative (*n* = 30)	Procedure Billing Codes not included in Data (*n* = 11) Incorrectly Classified as Herniated Disc (*n* = 8) CPT code (63046, 63267) not included in algorithm (*n* = 6) Incorrectly Marked as Non-Primary Indication because of collapsed vertebra diagnosis (*n* = 2) Incorrectly Marked as Non-Primary Indication because of fecal incontinence diagnosis (*n* = 1) Incorrectly Marked as Non-Primary Indication because of fracture diagnosis (*n* = 1) Incorrectly marked as Non-Primary Indication because of bladder dysfunction diagnosis (*n* = 1)
Knee Osteoarthritis (*n* = 11)	False Positive (*n* = 2)	Procedure was actually to revise previous knee replacement (*n* = 1) Rheumatoid Arthritis Case (*n* = 1)
	False Negative (*n* = 9)	Procedure Billing Codes not included in Data (*n* = 6) CPT code (27486, 27487) not included in algorithm (*n* = 2) Incorrectly Marked as Non-Primary Indication because of osteonecrosis diagnosis (*n* = 1)
Hip Osteoarthritis (*n* = 23)	False Positive (*n* = 2)	Osteonecrosis Diagnosis (*n* = 1) Developmental Hip Dysplasia Case (*n* = 1)
	False Negative (*n* = 21)	Incorrectly Marked as Non-Primary Indication because of osteonecrosis diagnosis (*n* = 12) Procedure Billing Codes not included in Data (*n* = 6) CPT code (27134) not included in algorithm (*n* = 1) Incorrectly Marked as Non-Primary Indication because of fracture diagnosis (*n* = 1) Incorrectly Marked as Non-Primary Indication because of neoplasm diagnosis (*n* = 1)

KOA cases were misclassified for a variety of reasons, including missing CPT codes. HOA procedures, on the other hand, were often misclassified because the algorithm identified a diagnosis of osteonecrosis, fracture, or bone neoplasm as the primary indication instead of HOA, even when the gold standard review indicated that HOA was the primary indication for surgery. This occurred because some of the ICD-10 codes included in the algorithm are not specific to a certain site (e.g., hip or lumbar spine). The HOA procedure group was the only group of patients where some of the exclusion ICD-10 codes dramatically decreased the sensitivity of the algorithm.

## Discussion

### Key findings

Determining the type of procedure performed and the primary indication for that procedure can be useful in a variety of contexts. In particular, it is important for studies that evaluate whether or not a decision to have surgery was the result of a SDM process, since SDM interventions are often only applicable for certain procedures and indications where there are multiple treatment options available [22, 23]. The algorithm developed in this study was able to classify surgical cases with the correct procedure and primary indication combination with high specificity across the four combinations analyzed. The sensitivity, however, varied significantly across these combinations. The sensitivity was high for the KOA group (> 0.9), medium for the SpS and HOA groups (0.75–0.9), and lower for the HD group (< 0.75).

### Implications

The primary utility of the algorithm developed in this paper is to automate the identification of patients for inclusion in research studies on orthopedic surgery used to treat hip or knee osteoarthritis, spinal stenosis, or herniated disc. It is especially useful for the identification of patients who are eligible for SDM and to facilitate the collection of SDM performance measures following surgery, since SDM is recommended for surgeries used to treat these conditions.

We identified varying sensitivity and specificity of the algorithm across these surgery/primary indication combinations, implying that the application of this algorithm may only be useful in certain situations. For instance, the high specificities of the final determinations indicate that, since the false positive rate is low, any surgical case that is marked by the algorithm with a particular surgery/primary indication combination could be reliably included in a study evaluating patients in that group. Similarly, the high sensitivity value for the KOA procedure group indicates that, since the false negative rate is low, a surgical case that is not marked as included in that group by the algorithm could be excluded from a study focusing on knee arthroplasty procedures to treat KOA without a high risk of missing a relevant patient. The lower sensitivity values for the HD, SpS, and HOA groups, on the other hand, means that there is a higher false negative rate and further manual review would be needed to decide whether or not patients should actually be excluded from a study if the algorithm marks them as not meeting the criteria for one of those groups. For the HOA group, this would be relatively straightforward, since most of the false negative classifications were made because the algorithm did not correctly classify HOA as the primary indication due to an exclusion diagnosis included in their record. Since the number of patients with those exclusion diagnoses is relatively small among the patients receiving the procedures included in the algorithm, a manual review of those cases would not necessarily be that costly. For the HD and SpS groups, however, such a manual review could be costly, since there was a relatively high number of false negatives for those groups in the validation dataset used in this study.

The usefulness of this algorithm for identifying KOA and HOA as the primary indication for arthroplasty procedures also represents a novel development compared to past research. Previous studies have validated algorithms to identify hip or knee arthroplasty procedures, such as Daneshvar, Forster and Dervin [12], but these studies generally only used ICD codes as part of the algorithm, as opposed to both ICD and CPT codes, and no previous studies could be found that validated an algorithm to identify the primary indication for hip or knee arthroplasty procedures using administrative data.

The use of the algorithm for the HD and SpS groups present more of a challenge, since most of those misclassifications occurred because the algorithm did not correctly discriminate between a HD and SpS diagnosis. Past studies such as Kazberouk et al. [9] have also encountered similar issues when using ICD-10 and CPT codes to distinguish between different spine diagnoses, suggesting that, in general, it is difficult to create automated algorithms that can reliably separate SpS and HD cases using ICD-10 and CPT codes. The algorithm developed in this study attempts to mitigate this issue by allowing some of the codes to overlap between the two diagnoses, and then applying an age cutoff where older patients are marked as SpS cases and younger patients are marked as HD cases. Other methods of discriminating the two diagnoses were tested, such as letting the SpS diagnosis “dominate” and marking a case with an SpS diagnoses whenever both SpS and HD were identified by the list of ICD-10 and CPT codes. None of these other methods, however, significantly changed the final sensitivity and specificity results of the algorithm. In general, this difficulty is likely rooted in the fact that these two diagnoses are sometimes not mutually exclusive, so administrative data will not necessarily have a consistent coding pattern for either condition. This inconsistency makes it difficult to develop an algorithm that can differentiate between the two when relying solely on this administrative data. In a research setting, correcting for this bias would require a chart review of each of the patients with an included spine procedure to determine their correct diagnosis, which could be costly in terms of the time and staff needed to conduct the review.

Still, the algorithm developed in this paper does represent an improvement over previously published algorithms to identify the indications for spine surgery. Cherkin et al. [15] did validate an algorithm to identify patients with “mechanical low back problems,” which generally reflects a certain set of primary indications for surgery including SpS and HD, but this study only used ICD-9 codes, rather than the current ICD-10 codes, is old enough that it may not reflect current coding practices, and does not attempt to differentiate between SpS and HD. Furthermore, other work has indicated that CPT codes, which Cherkin et al. [15] did not include in their algorithm, provide a greater level of detail about spine surgeries in administrative data [16]. Therefore, by explicitly focusing on the identification of elective procedures and by incorporating both ICD-10 and CPT codes, the algorithm developed in this study provides a useful updated method of identifying patients who have received spinal surgery to treat SpS or HD, even with the difficulty of differentiating between the two conditions.

It should also be noted that the usefulness of the algorithm as a whole may change depending on the characteristics of the surgical cases used as the base population. As shown in Fig. 1, as the prior probability increases that any given case out of that population matches the procedure/primary indication combination of interest, the positive predictive value of the algorithm increases (and vice versa for the negative predictive value). This means that utility of the algorithm will ultimately depend on the setting in which it is used, and could be used in combination with other screening methods that change the prior probability of the base population.

### Limitations

One primary limitation in validating this algorithm is the small number of spinal surgery cases with HD as the primary indication. As a result, the sensitivity for that procedure/indication combination had a wide confidence interval, making it difficult to determine if the algorithm is useful in that context. Another key limitation is that in rare cases the data for any given surgical patient is not complete (likely because the surgery procedures were recorded using a different billing system). In these cases, the accuracy of the algorithm would have been underestimated, compared to the performance of the algorithm if all the data had been available. In addition to this bias, it also highlights a major drawback to the use of administrative data in general to make determinations about the characteristics of surgical cases.

## Conclusions

By validating this algorithm against a gold standard of manual chart review, future researchers will be able to conduct more efficient and accurate analyses on elective orthopedic surgeries using administrative claims data. Future work to improve this type of algorithm should include finding ways to differentiate between SpS and HD indications using administrative data.

## Supplementary information

**Additional file 1.**

## Data Availability

The datasets generated and/or analyzed during the current study are not publicly available due to the fact that the data contain confidential medical records.

## Abbreviations

SDM: Shared Decision-Making
ICD: International Classification of Diseases
CPT: Current Procedural Terminology
KOA: Knee Osteoarthritis
HOA: Hip Osteoarthritis
SpS: Spinal Stenosis
HD: Herniated Disc
