# Supplementary material for: Validation of an electronic coding algorithm to identify the primary indication of orthopedic surgeries from administrative data
Source: BMC Med Inform Decis Mak. 2020 Aug 12;20:187. doi: 10.1186/s12911-020-01175-1 (PMC7425151; doi:10.1186/s12911-020-01175-1)
Supplement: Supplementary file 1 — Additional file 1. [file 12911_2020_1175_MOESM1_ESM.pdf]

Appendix

Supplemental material for: "Validation of an electronic coding algorithm to identify the primary indication of orthopedic surgeries from administrative data"

Descriptions of ICD-10 Codes Used in the Automated Algorithm

Code	Description
C41.2	Malignant neoplasm of vertebral column
C41.4	Malignant neoplasm of pelvic bones, sacrum and coccyx
C41.9	Malignant neoplasm of bone and articular cartilage, unspecified
C79.51	Secondary malignant neoplasm of bone
G83.4	Cauda equina syndrome
M16.0	Bilateral primary osteoarthritis of hip
M16.10	Unilateral primary osteoarthritis, unspecified hip
M16.11	Unilateral primary osteoarthritis, right hip
M16.12	Unilateral primary osteoarthritis, left hip
M16.2	Bilateral osteoarthritis resulting from hip dysplasia
M16.30	Unilateral osteoarthritis resulting from hip dysplasia unspecified hip
M16.31	Unilateral osteoarthritis resulting from hip dysplasia right hip
M16.32	Unilateral osteoarthritis resulting from hip dysplasia left hip
M16.4	Bilateral post-traumatic osteoarthritis of hip
M16.50	Unilateral post-traumatic osteoarthritis, unspecified hip
M16.51	Unilateral post-traumatic osteoarthritis, right hip
M16.52	Unilateral post-traumatic osteoarthritis, left hip
M16.6	Other bilateral secondary osteoarthritis of hip
M16.7	Other unilateral secondary osteoarthritis of hip
M16.9	Osteoarthritis of hip, unspecified
M17.0	Bilateral primary osteoarthritis of knee
M17.10	Unilateral primary osteoarthritis, unspecified knee
M17.11	Unilateral primary osteoarthritis, right knee
M17.12	Unilateral primary osteoarthritis, left knee
M17.2	Bilateral post-traumatic osteoarthritis of knee
M17.30	Unilateral post-traumatic osteoarthritis, unspecified knee
M17.31	Unilateral post-traumatic osteoarthritis, right knee
M17.32	Unilateral post-traumatic osteoarthritis, left knee
M17.4	Other bilateral secondary osteoarthritis of knee
M17.5	Other unilateral secondary osteoarthritis of knee
M17.9	Osteoarthritis of knee, unspecified
M47.16	Other spondylosis with myelopathy lumbar region
M47.26	Other spondylosis with radiculopathy, lumbar region
M47.27	Other spondylosis with radiculopathy, lumbosacral region
M48.00	Spinal stenosis, site unspecified

Code	Description
M48.061	Spinal stenosis, lumbar region without neurogenic claudication
M48.062	Spinal stenosis, lumbar region with neurogenic claudication
M48.07	Spinal stenosis, lumbar region lumbosacral region
M48.08	Spinal stenosis, lumbar region sacral and sacrococcygeal region
M48.46*	Fatigue fracture of vertebra, lumbar region
M48.56*	Collapsed vertebra, not elsewhere classified, lumbar region
M48.57*	Collapsed vertebra, not elsewhere classified, lumbosacral region
M51.06	Intervertebral disc disorders with myelopathy, lumbar region
M51.16	Intervertebral disc disorders with radiculopathy, lumbar region
M51.17	Intervertebral disc disorders with radiculopathy, lumbosacral region
M51.26	Other intervertebral disc displacement, lumbar region
M51.27	Other intervertebral disc displacement, lumbosacral region
M51.36	Other intervertebral disc degeneration, lumbar region
M51.37	Other intervertebral disc degeneration, lumbosacral region
M80*	Osteoporosis with current pathological fracture
M84.30*	Stress fracture, unspecified site
M84.359*	Stress fracture, hip, unspecified
M84.40*	Pathological fracture, unspecified site
M84.459*	Pathological fracture, hip, unspecified
M84.48*	Pathological fracture, other site
M84.50*	Pathological fracture in neoplastic disease, unspecified site
M84.559*	Pathological fracture in neoplastic disease, hip, unspecified
M84.58*	Pathological fracture in neoplastic disease, other specified site
M84.60*	Pathological fracture in other disease, unspecified site
M87*	Osteonecrosis
N31*	Neuromuscular dysfunction of bladder, not elsewhere classified
R15*	Fecal incontinence
R32	Unspecified urinary incontinence
S22.08*	Fracture of T11-T12 vertebra
S32.0*	Fracture of lumbar vertebra
S32.4*	Fracture of acetabulum
S72.0*	Fracture of head and neck of femur
S72.1*	Pertrochanteric fracture
S72.2*	Subtrochanteric fracture of femur

Descriptions of CPT Codes Used in the Automated Algorithm

Code	Description
22224	Osteotomy of spine, including discectomy, anterior approach, single vertebral segment; lumbar
22533	Arthrodesis, lateral extracavitary technique, including minimal discectomy to prepare interspace (other than for decompression); lumbar
22558	Arthrodesis, anterior interbody technique, including minimal discectomy to prepare interspace (other than for decompression); lumbar
22586	Arthrodesis, pre-sacral interbody technique, including disc space preparation, discectomy, with posterior instrumentation, with image guidance, includes bone graft when performed, L5-S1 interspace
22612	Arthrodesis, posterior or posterolateral technique, single level; lumbar (with lateral transverse technique, when performed)
22614	Arthrodesis, posterior or posterolateral technique, single level; each additional vertebral segment
22630	Arthrodesis, posterior interbody technique, including laminectomy and/or discectomy to prepare interspace (other than for decompression), single interspace; lumbar
22632	Arthrodesis, posterior interbody technique, including laminectomy and/or discectomy to prepare interspace (other than for decompression), single interspace; each additional interspace (list separately in addition to code for primary procedure)
22633	Arthrodesis, combined posterior or posterolateral technique with posterior interbody technique including laminectomy and/or discectomy sufficient to prepare interspace (other than for decompression), single interspace and segment; lumbar
22800	Arthrodesis, posterior, for spinal deformity, with or without cast; up to 6 vertebral segments
22802	Arthrodesis, posterior, for spinal deformity, with or without cast; 7 to 12 vertebral segments
22804	Arthrodesis, posterior, for spinal deformity, with or without cast; 13 or more vertebral segments
22808	Arthrodesis, anterior, for spinal deformity, with or without cast; 2 to 3 vertebral segments
22810	Arthrodesis, anterior, for spinal deformity, with or without cast; 4 to 7 vertebral segments
22857	Total disc arthroplasty (artificial disc), anterior approach, including discectomy to prepare interspace (other than for decompression), single interspace, lumbar
22862	Revision including replacement of total disc arthroplasty (artificial disc), anterior approach, single interspace; lumbar
22867	Insertion of interlaminar/interspinous process stabilization/distraction device, without fusion, including image guidance when performed, with open decompression, lumbar; single level
27125	Hemiarthroplasty, hip, partial (eg, femoral stem prosthesis, bipolar arthroplasty)
27130	Arthroplasty, acetabular and proximal femoral prosthetic replacement (total hip arthroplasty), with or without autograft or allograft
27132	Conversion of previous hip surgery to total hip arthroplasty, with or without autograft or allograft
27438	Arthroplasty, patella; with prosthesis
27440	Arthroplasty, knee, tibial plateau
27441	Arthroplasty, knee, tibial plateau; with debridement and partial synovectomy
27442	Arthroplasty, femoral condyles or tibial plateau(s), knee
27443	Arthroplasty, femoral condyles or tibial plateau(s), knee; with debridement and partial synovectomy

Code	Description
27445	Arthroplasty, knee, hinge prosthesis (eg, Walldius type)
27446	Arthroplasty, knee, condyle and plateau; medial OR lateral compartment
27447	Arthroplasty, knee, condyle and plateau; medial AND lateral compartments with or without patella resurfacing (total knee arthroplasty)
62287	Decompression procedure, percutaneous, of nucleus pulposus of intervertebral disc, any method utilizing needle based technique to remove disc material under fluoroscopic imaging or other form of indirect visualization, with the use of an endoscope, with discography and/or epidural injection(s) at the treated level(s), when performed, single or multiple levels, lumbar
63005	Laminectomy with exploration and/or decompression of spinal cord and/or cauda equina, without facetectomy, foraminotomy or discectomy (eg, spinal stenosis), 1 or 2 vertebral segments; lumbar, except for spondylolisthesis
63011	Laminectomy with exploration and/or decompression of spinal cord and/or cauda equina, without facetectomy, foraminotomy or discectomy (eg, spinal stenosis), 1 or 2 vertebral segments; sacral
63012	Laminectomy with removal of abnormal facets and/or pars inter-articularis with decompression of cauda equina and nerve roots for spondylolisthesis, lumbar (Gill type procedure)
63017	Laminectomy with exploration and/or decompression of spinal cord and/or cauda equina, without facetectomy, foraminotomy or discectomy (eg, spinal stenosis), more than 2 vertebral segments; lumbar
63030	Laminotomy (hemilaminectomy), with decompression of nerve root(s), including partial facetectomy, foraminotomy and/or excision of herniated intervertebral disc; 1 interspace, lumbar
63035	Laminotomy (hemilaminectomy), with decompression of nerve root(s), including partial facetectomy, foraminotomy and/or excision of herniated intervertebral disc; each additional interspace, cervical or lumbar (List separately in addition to code for primary procedure)
63042	Laminotomy (hemilaminectomy), with decompression of nerve root(s), including partial facetectomy, foraminotomy and/or excision of herniated intervertebral disc, reexploration, single interspace; lumbar
63047	Laminectomy, facetectomy and foraminotomy (unilateral or bilateral with decompression of spinal cord, cauda equina and/or nerve root(s), (eg, spinal or lateral recess stenosis)), single vertebral segment; lumbar
63048	Laminectomy, facetectomy and foraminotomy (unilateral or bilateral with decompression of spinal cord, cauda equina and/or nerve root(s), (eg, spinal or lateral recess stenosis)), single vertebral segment; each additional segment, cervical, thoracic, or lumbar (List separately in addition to code for primary procedure)
63056	Transpedicular approach with decompression of spinal cord, equina and/or nerve root(s) (eg, herniated intervertebral disk), single segment; lumbar (including transfacet, or lateral extraforaminal approach) (eg, far lateral herniated intervertebral disk)
63087	Vertebral corpectomy (vertebral body resection), partial or complete, combined thoracolumbar approach with decompression of spinal cord, cauda equina or nerve root(s), lower thoracic or lumbar; single segment
63088	Vertebral corpectomy (vertebral body resection), partial or complete, combined thoracolumbar approach with decompression of spinal cord, cauda equina or nerve root(s), lower thoracic or lumbar; each additional segment (List separately in addition to code for primary procedure)

Code	Description
63090	Vertebral corpectomy (vertebral body resection), partial or complete, transperitoneal or retroperitoneal approach with decompression of spinal cord, cauda equina or nerve root(s), lower thoracic, lumbar, or sacral; single segment
63091	Vertebral corpectomy (vertebral body resection), partial or complete, transperitoneal or retroperitoneal approach with decompression of spinal cord, cauda equina or nerve root(s), lower thoracic, lumbar, or sacral; each additional segment (List separately in addition to code for primary procedure)
63102	Vertebral corpectomy (vertebral body resection), partial or complete, lateral extracavitary approach with decompression of spinal cord and/or nerve root(s) (eg, for tumor or retropulsed bone fragments); lumbar, single segment
63103	Vertebral corpectomy (vertebral body resection), partial or complete, lateral extracavitary approach with decompression of spinal cord and/or nerve root(s) (eg, for tumor or retropulsed bone fragments); thoracic or lumbar, each additional segment (List separately in addition to code for primary procedure)
63170	Laminectomy with myelotomy (eg, Bischof or DREZ type), cervical, thoracic, or thoracolumbar

CPT Copyright 2017 American Medical Association. All rights reserved. CPT® is a registered trademark of the American Medical Association.

Two-by-Two Tables for Identification of Condition as an Indication for Surgery by the Algorithm

Hip Arthroplasty with Hip OA Indication

		Gold Standard Determination		
		Hip Arthroplasty w/ Hip OA	Not Hip Arthroplasty w/ Hip OA	Total
Algorithm Determination	Hip Arthroplasty w/ Hip OA	133	38	171
	Not Hip Arthroplasty w/ Hip OA	1	258	259
	Total	134	296	430

Knee Arthroplasty with Knee OA Indication

		Gold Standard Determination		
		Knee Arthroplasty w/ Knee OA	Not Knee Arthroplasty w/ Knee OA	Total
Algorithm Determination	Knee Arthroplasty w/ Knee OA	114	49	163
	Not Knee Arthroplasty w/ Knee OA	4	263	267
	Total	118	312	430

Lumbar Spinal Surgery with SpS Indication

		Gold Standard Determination		
		Lumbar Spinal Surgery w/ SpS	Not Lumbar Spinal Surgery w/ SpS	Total
Algorithm Determination	Lumbar Spinal Surgery w/ SpS	119	48	167
	Not Lumbar Spinal Surgery w/ SpS	12	226	238
	Total	131	274	405

Lumbar Spinal Surgery with HD Indication

		Gold Standard Determination		
		Lumbar Spinal Surgery w/ HD	Not Lumbar Spinal Surgery w/ HD	Total
Algorithm Determination	Lumbar Spinal Surgery w/ HD	52	91	143
	Not Lumbar Spinal Surgery w/ HD	2	260	262
	Total	54	351	405

Two-by-Two Tables for Identification of Condition as the Primary Indication for Surgery by the Algorithm

Hip Arthroplasty with Hip OA Primary Indication

		Gold Standard Determination		
		Hip Arthroplasty w/ Hip OA	Not Hip Arthroplasty w/ Hip OA	Total
Algorithm Determination	Hip Arthroplasty w/ Hip OA	107	2	109
	Not Hip Arthroplasty w/ Hip OA	21	300	321
	Total	128	302	430

Knee Arthroplasty with Knee OA Primary Indication

		Gold Standard Determination		
		Knee Arthroplasty w/ Knee OA	Not Knee Arthroplasty w/ Knee OA	Total
Algorithm Determination	Knee Arthroplasty w/ Knee OA	105	2	107
	Not Knee Arthroplasty w/ Knee OA	9	314	323
	Total	114	316	430

Lumbar Spinal Surgery with SpS Primary Indication

		Gold Standard Determination		
		Lumbar Spinal Surgery w/ SpS	Not Lumbar Spinal Surgery w/ SpS	Total
Algorithm Determination	Lumbar Spinal Surgery w/ SpS	97	16	113
	Not Lumbar Spinal Surgery w/ SpS	30	262	292
	Total	127	278	405

Lumbar Spinal Surgery with HD Primary Indication

		Gold Standard Determination		
		Lumbar Spinal Surgery w/ HD	Not Lumbar Spinal Surgery w/ HD	Total
Algorithm Determination	Lumbar Spinal Surgery w/ HD	37	13	50
	Not Lumbar Spinal Surgery w/ HD	16	339	355
	Total	53	352	405
